# Variants in *BRWD3* associated with X‐linked partial epilepsy without intellectual disability

**DOI:** 10.1111/cns.14057

**Published:** 2022-12-13

**Authors:** Mao‐Qiang Tian, Xiao‐Rong Liu, Si‐Mei Lin, Jie Wang, Sheng Luo, Liang‐Di Gao, Xiao‐Bin Chen, Xiao‐Yu Liang, Zhi‐Gang Liu, Na He, Yong‐Hong Yi, Wei‐Ping Liao, For the China Epilepsy Gene 1.0 Project

**Affiliations:** ^1^ Institute of Neuroscience and Department of Neurology of the Second Affiliated Hospital of Guangzhou Medical University Key Laboratory of Neurogenetics and Channelopathies of Guangdong Province and the Ministry of Education of China Guangzhou China; ^2^ Department of Pediatrics Affiliated Hospital of Zunyi Medical University Zunyi China; ^3^ Department of Pediatrics The 900th Hospital of Joint Logistic Support Force Fuzhou China; ^4^ Department of Pediatrics, Affiliated Foshan Maternity & Child Healthcare Hospital Southern Medical University Foshan China

**Keywords:** *BRWD3* gene, epilepsy, intellectual disability, whole‐exome sequencing

## Abstract

**Aims:**

Etiology of the majority patients with idiopathic partial epilepsy (IPE) remains elusive. We thus screened the potential disease‐associated variants in the patients with IPE.

**Methods:**

Trios‐based whole exome sequencing was performed in a cohort of 320 patients with IPE. Frequency and molecular effects of variants were predicted.

**Results:**

Three novel *BRWD3* variants were identified in five unrelated cases with IPE, which were four male cases and one female case. The variants included two recurrent missense variants (c.836C>T/p.Thr279Ile and c.4234A>C/p.Ile1412Leu) and one intronic variant close to splice site (c.2475 + 6A>G). The two missense variants were located in WD40 repeat domain and bromodomain, respectively. They were predicted to be damaging by silico tools and change hydrogen bonds with surrounding amino acids. The frequency of mutant alleles in this cohort was significantly higher than that in the controls of East Asian and all population of gnomAD. All these variants were inherited from the asymptomatic mothers. Four male cases presented frequent seizures at onset, while the female case only had two fever‐triggered seizures. They showed good responses to valproate and lamotrigine, then finally became seizure free. All the cases had no intellectual disability. Further analysis demonstrated that all previously reported destructive variants of *BRWD3* caused intellectual disability, while missense variants located in WD40 repeat domains and bromodomains of *BRWD3* were associated with epilepsy.

**Conclusion:**

*BRWD3* gene is potentially associated with X‐linked partial epilepsy without intellectual disability. The genotypes and locations of *BRWD3* variants may explain for their phenotypic variation.

## Graphical abstract

This study reports three novel variants of *BRWD3* in five unrelated cases with partial epilepsy and without mental retardation and demonstrates that the *BRWD3* gene is potentially a candidate pathogenic gene of epilepsy. The genotypes and locations of *BRWD3* variants may explain for their phenotypic variation.

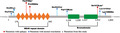

## 1 INTRODUCTION

*BRWD3* (OMIM* 300553), mapping to Xq21.1, encodes bromodomain and WD‐repeat domain‐containing protein 3 (*BRWD3*). *BRWD3* is a protein with 1802 amino‐acids, containing WD40 repeat domains in N‐terminal and bromodomains in C‐terminal. This protein is epigenetic reader of histone acetylation that regulates chromatin remodeling, ubiquitination, and signal transduction.^1^, ^2^, ^3^ As a regulator of the JAK/STAT pathway, *BRWD3* may affect the cellular proliferation and level of brain‐derived neurotrophic factor and γ‐aminobutyric acid type A receptor.^4^, ^5^ *BRWD3* is ubiquitously expressed (including in the brain) and predominantly expressed in the embryonic period. Hemizygous deficiency of *BRWD3* mice exhibited embryonic growth retardation and microcephaly. Previously, variants in *BRWD3* have been associated with mental retardation, X‐linked 93 (MRX93, OMIM# 300659).^1^ However, the link between *BRWD3* and epilepsy remains unknown. Here, we performed trios‐based whole exome sequencing (WES) in a cohort of 320 patients with unexplained partial (focal) epilepsy without acquired causes. Three *BRWD3* variants were identified in five unrelated cases. Further analysis demonstrated that previously reported destructive mutations of *BRWD3* were associated with intellectual disability, while the missense variants in WD40 repeat domains and bromodomains were associated with epilepsy.

## 2 MATERIALS AND METHODS

### 2.1 Subjects

A total of 320 patients (192 males and 128 females) with unexplained partial epilepsy were recruited from the Epilepsy Center of the Second Affiliated Hospital of Guangzhou Medical University between July 2012 and March 2020. The studies adhered to the guidelines of the International Committee of Medical Journal Editors with regard to patients consent for research or participation. This study was approved by the ethics committee of the Second Affiliated Hospital of Guangzhou Medical University (Approval ethics number: 2020‐hs‐49). All participants gave written informed consents.

Clinical data of affected individuals were obtained through face‐to‐face review by at least one of the authors. Detailed clinical information of the patients were collected, including age, gender, types and frequencies of seizures, general and neurological examination results, family history, and response to anti‐epileptic drugs. Neuroimaging scans including magnetic resonance imaging (MRI) were performed to detect any brain structure abnormalities. Long‐term video‐electroencephalography (EEG) monitoring records that included hyperventilation, intermittent photic stimulation, open‐close eyes test, and sleeping recording were conducted. Epileptic seizures and epilepsies were diagnosed according to the criteria of the Commission on Classification and Terminology of the ILAE (1981, 1989, 2001, 2010, and 2017).^6^, ^7^, ^8^ Epilepsies with acquired causes were excluded.

### 2.2 Trios‐based WES

Blood samples were obtained from the probands, their parents and other family members (if available) to determine the origin of the identified genetic variants. Genomic DNA was extracted from peripheral blood using a QuickGene DNA whole blood kit (Fujifilm). Trios‐based WES was conducted on the Illumina HiSeq 2500/4000 platform by BGI‐Shenzhen as previously reported (Wang et al., 2018; Shi et al., 2019). A case‐by‐case analytical approach were performed to identify candidate causative variants in each trio. We first prioritized the rare variants with a minor allele frequency <0.005 in the 1000 Genomes Projects, Exome Aggregation Consortium, and gnomAD. Potentially pathogenic variants including frameshift, nonsense, canonical splice site, initiation codon, and missense variants predicted as being damaging by in silico tools (http://varcards.biols.ac.cn/) were retained. We then screened the potential disease‐causing variants in each case under five models: (1) epilepsy‐associated gene variants, (2) de novo dominant variants, (3) autosomal recessive inheritance model, (4) X‐linked model, and (5) co‐segregated variants. Possible novel epilepsy genes were considered if a gene presented recurrent de novo variants, biallelic variants, hemizygous variants, and variants with segregations. We aimed to discover novel potential epilepsy genes, so known epilepsy‐associated genes were excluded^9^ in the analysis of the present study. Conservation of mutated residues were evaluated using sequence alignment of phylogenetic species. Sanger sequencing were performed for confirming potential clinical significance variants. All variants were annotated based on the transcript NM_153252.

### 2.3 Statistical analysis

SPSS Statistics 26.0 was used for statistical analysis. The frequencies of the variants in candidate gene in the present cohort were compared with that in the control populations by Fisher's exact test (R4.0.2).^10^ A *p* value of <0.05 was considered to be statistically significant.

### 2.4 Mutation analysis

Protein modeling was performed to predict the effects of missense variants on molecular structure by the Iterative Threading ASSEmbly Refinement (I‐TASSER) software. The confidence of each modeling was quantitatively measured by a C‐score in the range of [−5, 2]. PyMOL 1.7 was used for three‐dimensional protein structure visualization and analysis. I‐Mutant server was used for prediction of protein stability changes. The changes of the protein stability were assessed using the free energy stability change (DDG, Kcal/mol) value.

In an attempt to evaluate the genotype–phenotype correlation, we systematically reviewed *BRWD3* mutations on the PubMed database till March 2021, and mutations in patients with detailed neurological phenotypes were analyzed.

## 3 RESULTS

### 3.1 Identification of *BRWD3* variants

**FIGURE 1:**
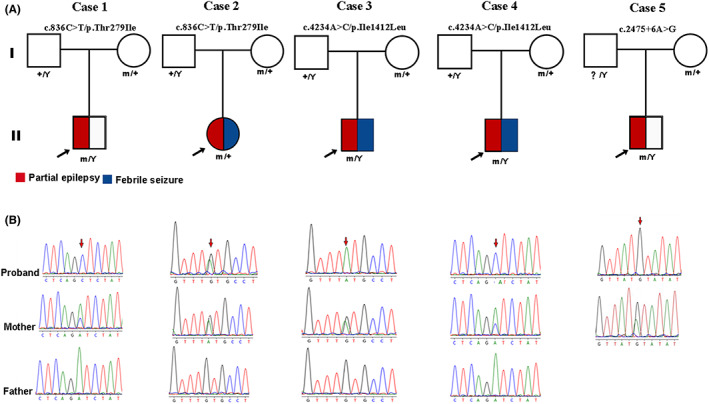
Genetic data on the patients with *BRWD3* variants. (A) Pedigrees of the five cases with *BRWD3* variants and their corresponding phenotypes. (B) DNA sequence chromatograms of the *BRWD3* variant. Arrows indicate the positions of the variants

Among the 320 patients with unexplained partial epilepsy, three *BRWD3* variants were identified in five unrelated cases (Figure 1 and Table 1), including two recurrent missense variants (c.836C>T/p.Thr279Ile and c.4234A>C/p.Ile1412Leu) and one intronic variant close to splice site (c.2475 + 6A>G). The five cases had no other pathogenic or likely pathogenic variants in genes known to be associated with seizure disorders.^9^ All the variants were inherited from their asymptomatic mothers.

**TABLE 1:** Clinical features of the individuals with *BRWD3* variants

Cases	Variants (NM_153252)	Gender	Present age	Diagnosis	FS/age	Onset of aFS	Seizure & frequency	Effective AEDs	ID	EEG	Brain MRI	Outcomes
1	c.836C>T; p. Thr279Ile	Male	11 yrs	PE	–	9 yrs	CPS, 7–8 times/day	LTG	No	Ictal: left frontal onset; Interictal: left frontal discharges	Normal	Seizure free for 1.5 yrs
2	c.836C>T; p. Thr279Ile	Female	5 yrs	PE + FS	+/4.5 yrs	–	Two focal seizures triggered by fever	LTG	No	Right rolandic discharges	Normal	Seizure free for 6 mons
3	c.4234A>C; p. Ile1412Leu	Male	16 yrs	PE + FS	+/5 yrs	9 yrs	CPS, 10–15 times/mon	VPA, LTG	No	NA	Normal	Seizure free for 6 mons
4	c.4234A>C; p. Ile1412Leu	Male	8 yrs	PE + FS	+/7 mons	10 mons	CPS, 1–2 times/mon	VPA	No	Right frontal discharges	Normal	Seizure free for 7 yrs
5	c.2475 + 6A>G	Male	13 yrs	PE	–	12 yrs	CPS, 1–2 times/mon	VPA	No	Discharges in right hemisphere	Normal	Seizure free for 1 yr

Abbreviations: aFS, afebrile seizure; ASMs, anti‐seizure medicines; CBZ, carbamazepine; CPS, complex partial seizure; EEG, electroencephalogram; ID, intellectual disability; LTG, lamotrigine; mon, month; MRI, magnetic resonance imaging; PE, partial epilepsy; VPA, valproate; yr, year.

**FIGURE 2:**
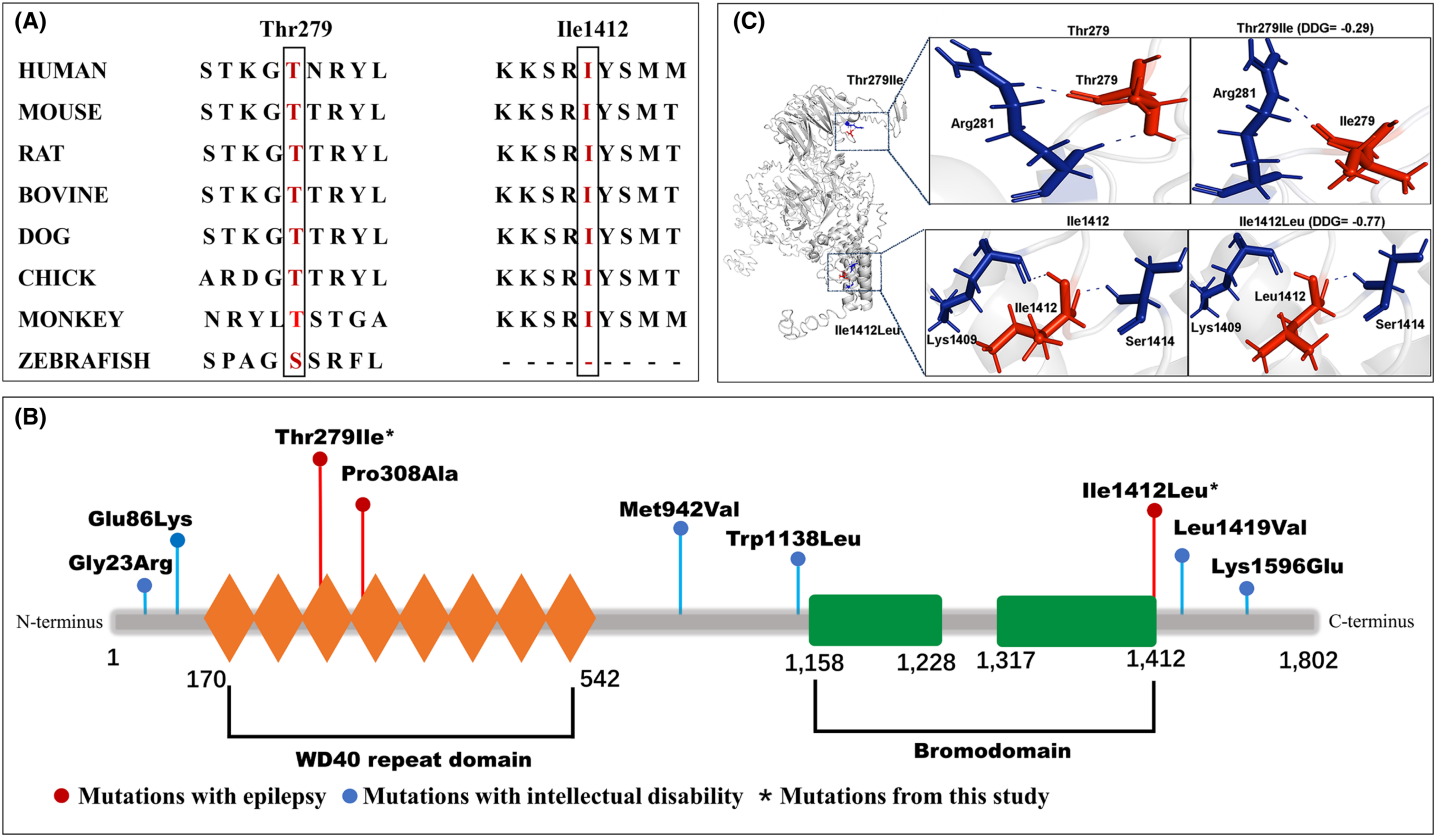
Schematic illustration of *BRWD3* variants. (A) Phylogenetic conservation of the Thr279 and Ile1412 (highlighted in red). These residues were conserved between species during evolution. (B) Schematic diagram of *BRWD3* and the localization of the missense variants of *BRWD3* identified in previous reports and in this study. (C) Hydrogen bond changes of mutants Thr279Ile and Ile1412Leu

The amino acid residues of the two missense variants are highly conserved in various species (Figure 2A). The two missense variants were suggested to be damaging by at least three silico tools (Table S1). Variant Thr279Ile was predicted to have more severe effects than variant Ile1412Leu according to Grantham scores (89 v.s. 5). The three variants are not present or at low minor allele frequency (MAF) in gnomAD database (Table 2). A statistical analysis on the frequency of *BRWD3* mutant alleles in this cohort was significantly higher than that in the controls of all population and East‐Asian population of gnomAD (5/448 vs. 12/76827 in controls of all populations, *p* = 3.74 × 10^−8^; v.s. 12/6670 in controls of East‐Asian population, *p* = 3.17 × 10^−3^; and 4/192 vs. 3/1906 hemizygotes in controls of East Asian populations, *p* = 1.91 × 10^−3^) (Table 2).

**TABLE 2:** Gene‐based burden analysis for *BRWD3* variants identified in this study

Variant (NM_153252.4)	Allele count/number from this study		Allele count/number in populations of gnomAD				
	All alleles	Hemizygote	All populations	Controls of all populations	East Asian populations	Controls of East Asian populations	Hemizygote in controls of East Asian populations
c.836C>T; p. Thr279Ile	2/448 (0.00446)	1/192 (0.00520)	5/174894 (2.86 × 10^−5^)	3/76827 (3.90 × 10^−5^)	5/13235 (3.78 × 10^−4^)	3/6670 (4.50 × 10^−4^)	1/1906 (5.24 × 10^−4^)
c.4234A>C; p. Ile1412Leu	2/448 (0.00446)	2/192 (0.0104)	17/203636 (8.35 × 10^−5^)	9/87142 (1.03 × 10^−4^)	17/14842 (0.00115)	9/7512 (0.00120)	2/2368 (8.38 × 10^−4^)
c.2475 + 6 A>G	1/448 (0.00223)	1/192 (0.00520)	−/−	−/−	−/−	−/−	−/−
Total	5/448 (0.0111)	4/192 (0.0208)	22/174894 (1.26 × 10^−4^)	12/76827 (1.56 × 10^−4^)	22/13235 (0.00166)	12/6670 (0.00180)	3/1906 (0.00157)
*p* value^+^			8.207 × 10^−9^	3.742 × 10^−8^	1.63 × 10^−3^	3.17 × 10^−3^	1.91 × 10^−3^
OR (95% CI)			89.693 (26.39–243.37)	72.21 (18.84–221.56)	6.776 (1.99–18.44)	6.259 (1.72–19.19)	13.460 (2.259–92.500)

*Note*: *p* values and odds ratio were estimated with a 2‐sided Fisher's exact test.

Abbreviations: CI, confidence interval; gnomAD, Genome Aggregation Database; OR, odds ratio.

### 3.2 Clinical information

All probands showed infancy or childhood‐onset partial epilepsy, the onset age ranged from 7 months to 12 years. The main clinical features of the cases with *BRWD3* variant were summarized in Table 1. All patients were born to non‐consanguineous parents after an uneventful pregnancy. Intellectual and motor development were normal for all patients. Brain MRI in all the patients were normal.

**FIGURE 3:**
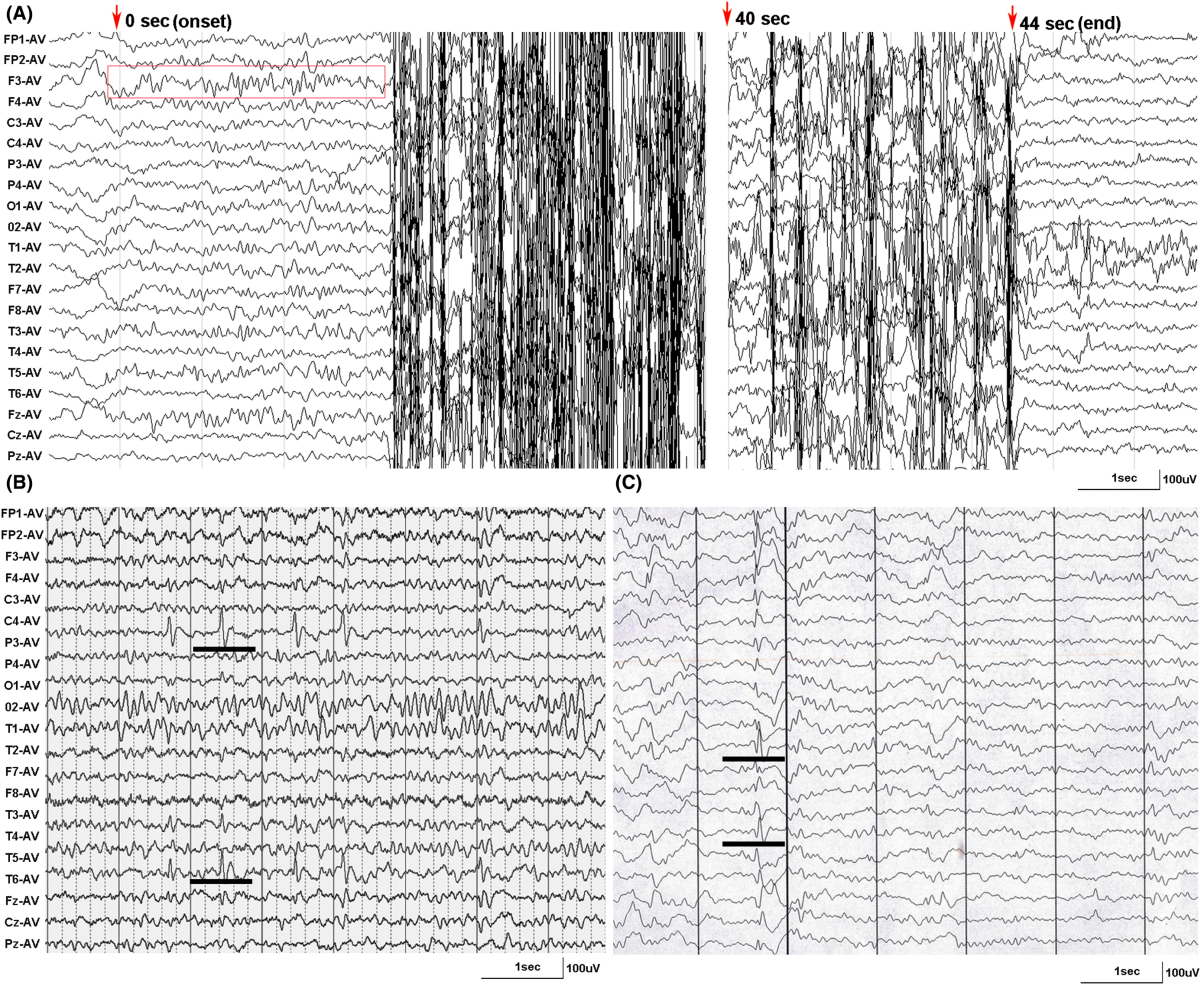
Ictal and interictal EEG in the cases with *BRWD3* variants. (A) EEG of case 1 detected partial seizures originated from left frontal region (red box indexed the seizure onset) (obtained at age of 9). (B) EEG of case 2 showed right centrotemporal discharges (black line) (at the age of 4.5 years). (C) EEG of case 5 indicated right temporal spike/sharp‐slow waves (black line) (obtained at age of 12 years)

Case 1 and case 2 harbored variant Thr279Ile. Case 1 was an 11‐year‐old boy. He had developed frequent nocturnal complex partial seizures (CPS) at frequency of 7–8 times daily since 9 years old. EEG recorded interictal left frontotemporal discharges and multiple attacks originated from left frontal region (Figure 3A). He got seizure free for 1.5 year with the treatment of lamotrigine (5 mg/kg/day). Case 2 is a 5‐year‐old girl. She had only two partial seizures triggered by fever when she was 4.5 years old. EEG showed right centrotemporal sharp‐slow waves (Figure 3B), so that lamotrigine (2.7 mg/kg/day) was given.

Variant Ile1412Leu was identified in two boys (case 3 and case 4). Case 3 experienced one febrile seizure (FS) at the age of 5 years. Subsequently, he experienced CPS at frequency of 10–15 times monthly since age of 9 years. He became seizure‐free with valproate (25 mg/kg/day) and lamotrigine (2.5 mg/kg/day). Case 4 presented one FS at age of 7 months. At the age of 10 months, he experienced twice CPS per month. Discharges in right frontal region were observed in EEG recordings. Valproate (35 mg/kg/day) was given since age of 11 months. He had been seizure‐free for 7 years.

Case 5 harbored variant c.2475 + 6A>G. This boy started to have partial seizures 1–2 times monthly at the age of 12 years. Interictal EEG indicated right temporal spike/sharp‐slow waves (Figure 3C). His seizures did not respond to carbamazepine (100 mg/day). He finally got seizure‐free after combining with valproate (750 mg/day). His father was unwilling to undergo genetic testing.

The five patients had partial seizures accompanied with focal discharges in EEGs. Except for the girl with only two fever‐triggered seizures (case 2), all the patients had frequent seizures at onset (ranged from several times a month to several times per day). However, all the patients showed good response to valproate and lamotrigine, then finally became seizure free.

### 3.3 Structural alteration of *BRWD3* protein

As shown schematically in Figure 2B, *BRWD3* successively contains WD40 repeat domains and bromodomains. Structural model of BWRD3 indicated residues 170–542 were located within the WD40 repeat domains, and residues 1158–1412 were located in bromodomains. The two missense variants were located in crucial domains, of which variant Thr279Ile was located in WD40 repeat domain, and variant Ile1412 was located in bromodomain.

I‐Mutant server was used to analyze the molecular effects of the missense variants. Both variants changed the hydrogen bonds (Figure 2C). Residue Thr279 originally formed two hydrogen bonds with Arg281. When threonine was replaced by isoleucine at residue Thr279, one hydrogen bond was disappeared. Residue Ile1412 originally formed hydrogen bonds with Lys1409 and Ser1414, respectively. When isoleucine was replaced by leucine at residue Ile1412, the hydrogen bond with Lys1409 was destroyed. Missense variants Thr279Ile and Ile1412Leu were predicted to be least stable with DDG value of −0.29 and −0.77, respectively (Figure 2C).

### 3.4 Genotype–phenotype correlation of *BRWD3* variants

To explore the mechanism underlying phenotypic variations, we analyzed genotype–phenotype associations in all reported *BRWD3* mutations with detailed neurological phenotypes. Previously, 32 mutations have been reported, including 21 destructive mutations (nine nonsense mutations, six frame shift mutations, three splice site mutations, and three gross deletions), seven missense mutations, one intronic mutation, and three gross duplications.^1^, ^11^, ^12^, ^13^, ^14^, ^15^, ^16^, ^17^, ^18^, ^19^, ^20^, ^21^, ^22^, ^23^, ^24^, ^25^, ^26^ Their clinical and molecular details were listed in Appendix S1 (Table S1). Further analysis demonstrated that all individuals who carrying destructive mutations were associated with intellectual disability. Among the missense mutations, six reported mutations associated with intellectual disability were located outside the WD40 repeat domains and bromodomains, while three mutations associated with epilepsy (one previously reported variant Pro308Ala^16^ and two variants identified in this study) were located in WD40 repeat domains and bromodomains (Figure 2B). Both two intronic mutations (c.1877‐5T>C^18^ and c.2475 + 6A>G) were associated with epilepsy. As for gross duplications, two patients carrying larger duplications had intellectual disability, and the patient carrying a smaller duplication (involving bromodomains) had Rolandic epilepsy.

## 4 DISCUSSION

*BRWD3* contains eight WD40 repeat domains and two bromodomains, which plays a crucial role in ubiquitination, brain development, and synaptic plasticity.^1^, ^12^, ^14^ This protein is predominantly expressed in the early stages of human development, especially in blastocyst. In animal model, hemizygous deficiency of *BRWD3* mice exhibited microcephaly and developmental delay. These evidences indicate that *BRWD3* is essential for normal development of central nervous system. *BRWD3* is highly expressed in human brain. Clinically, *BRWD3* mutations have been identified as the cause of MRX93 in human. However, the association between *BRWD3* mutations and epilepsy remains unknown. In the present study, we identified *BRWD3* variants in five unrelated cases with partial epilepsy. The variants included two recurrent missense variants and one intronic variant close to splice site. These variants had no or low allele frequency in the gnomAD database. Their frequency of MAF was significantly higher in the present cohort than that in control populations in gnomAD. The two missense variants were predicted to be damaging by multiple in silico tools and altered the protein conformation. Taking together the evidence that *BRWD3* gene is expressed in brain and associated with neurodevelopment, the *BRWD3* gene is suggested to be potentially a candidate pathogenic gene of partial epilepsy.

Knockout of *BRWD3* in mice exhibited short tail buds, microcephaly and, in some cases, embryonic growth retardation. Clinically, all destructive mutations of *BRWD3* caused MRX93; while missense mutations, intronic variants close to splice sites, and small gross duplications were potentially associated with epilepsy with infancy or childhood onset, suggesting a genotype–phenotype correlation. In previously reported studies, the patients with variants p.P308A, c.1877‐5T>C, and duplication of 155 kb including part of *BRWD3* gene presented seizures started at the age of 19 months, 2.2 years, and 2 years, respectively,^16^, ^18^, ^20^ which were consistent with the onset age of the patients in this study. Generally, missense mutations and intronic mutations close to splice sites led to relatively mild damage effect comparing to destructive mutations. In this study, all male cases had frequent seizures, while the female individuals carrying *BRWD3* variants were asymptomatic or had only rare fever‐triggered seizures, reflecting relatively moderate decrease or low dose dependence of *BRWD3*, and potentially explaining the incomplete penetrance. Additionally, the small gross duplication had milder symptoms than the large gross duplications. These evidences indicated the quantitative correlation between the degree of functional damage and the phenotypic severity, which supports the potential pathogenicity of *BRWD3* variants.

Our previous study showed that molecular sub‐regional location of the missense mutations was a critical factor to determine the pathogenicity of variants and associated with phenotypic severity. In this study, all missense variants associated with epilepsy (Thr279Ile, Pro308Ala, and Ile1412Leu) clustered in WD40 repeat domains or bromodomains, while the missense mutations associated with intellectual disability were located outside WD40 repeat domains and bromodomains, suggesting a molecular sub‐regional effect of *BRWD3* mutations. Bromodomains are protein interaction modules that specifically recognize ε‐N‐lysine acetylation motifs, a key event in the reading process of epigenetic signaling marks. There is strong evidence that epigenetic signaling, which exerts high fidelity regulation of gene expression and plays a crucial role in the pathophysiology of epileptogenesis and epilepsy.^27^ Bromodomains in the human genome include 61 subtypes and cluster into eight families (I–VIII) based on structure/sequence similarity. The human *BRD2* gene, family II of bromodomain proteins, has been associated with myoclonic epilepsy and photosensitive epilepsy with electroencephalographic abnormalities.^28^, ^29^ Disruption of *Brd2* in the mouse revealed that homozygous mutation (*Brd2* ^*−/−*^) is incompatible with life, while the heterozygotes increased susceptibility to provoked seizures and significantly decreased GABA markers.^30^ *BRWD3* belongs to the family III of bromodomain proteins and is similar to BRD2 in the structure and functions of bromodomain region. In addition, *BRWD3* also contains another crucial domain‐‐WD40 repeat domain, which has been reported to be involved in signal transduction and cell division.^3^ Previously, genes containing the WD repeat domains such as *WDR37* and *WDR45* have been reported to be associated with epilepsy.^31^, ^32^ In the present study, the missense variants associated with epilepsy were located in WD40 repeat domain or bromodomain. These evidences suggest that WD40 repeat domains and bromodomains of *BRWD3* may play a crucial role in the pathophysiology of epileptogenesis by affecting cell signal transduction, epigenetic signaling reading, level of neurotransmitters, and neurovascular changes.^33^, ^34^ Further studies on the pathological consequences/neurological dysfunction are required to elucidate the essential link between the *BRWD3* variants and epilepsy.

In the present study, all the patients with *BRWD3* variants had partial seizures accompanied with frontal or temporal discharges, which is anatomically consistent with predominant expression of *BRWD3* in the frontal cortex and hippocampus (www.proteinatlas.org/search/BRWD3). Although most patients showed frequent partial epilepsy in the early stage of onset, they all finally became seizure‐free with the treatment of valproate and/or lamotrigine. These evidences may provide a reference for clinical management of the patients with *BRWD3* mutations.

In this study, three *BRWD3* variants were identified in five unrelated cases with partial epilepsy. The mutation rate of *BRWD3* in this cohort was 1.56% (5/320). So far, over a thousand of genes are associated with epilepsy. However, the mutation rate in single gene is very low in patients with partial epilepsy. For example, *DEPDC5* variants were identified in 0.9% (2/220) cases with partial epilepsy^35^; *UNC13B* variants were in 1.8% (8/446) cases with partial epilepsy^36^; and *AFF2* in 1.3% (5/372) of the cases with partial epilepsy.^37^ Overall, the frequency of variants for each gene in the patients with partial epilepsy warrants further investigation.

This study has several limitations. The direct functional effects of the variants were not examined. Thus, the concrete biological basis for *BRWD3* variants‐associated seizures warrants further investigation. The total number of patients with *BRWD3* variants was relatively small in this study. More cases are needed to elucidate genotype–phenotype correlation of *BRWD3* gene in future studies.

## 5 CONCLUSIONS

In conclusion, this study identified three novel variants of *BRWD3* (including two recurrent missense variants) in the patients with partial epilepsy with favorable outcome and without intellectual disability. The frequency of mutant alleles in this cohort was significantly higher than that in the controls of East Asian and all population of gnomAD. Two missense variants were predicted to be damaging and alter the protein configuration. Taking together the evidence that *BRWD3* is expressed in brain and essential for normal neurodevelopment, *BRWD3* is suggested to be potentially a candidate pathogenic gene of epilepsy. The genotype and variant location help explaining the phenotypic variation.

## AUTHOR CONTRIBUTIONS

Mao‐Qiang Tian, Xiao‐Rong Liu and Si‐Mei Lin collected the data from patients and wrote the paper; Jie Wang analyzed genetic pathogenicity; Sheng Luo performed statistical analysis; Liang‐Di Gao performed whole exome sequencing data analysis; Xiao‐Bin Chen, Xiao‐Yu Liang, Zhi‐Gang Liu and Yong‐Hong Yi analyzed EEG recordings and neuro‐imaging data; Wei‐Ping Liao designed and supervised the study.

## FUNDING INFORMATION

This work was supported by grants from the National Natural Science Foundation of China (grant Nos. 81871015 and 81870903), Natural Science Foundation of Guangdong Province (2020A1515010108); Science and Technology Project of Guangzhou (Grant No. 201904020028); Science and Technology Project of Guangdong Province (Grant Nos. 2017B090904036 and 2017B030314159); Multi‐center Clinical Research Fund Project of the Second Affiliated Hospital of Guangzhou Medical University (2020‐LCYJ‐DZX‐03 and 2021‐LCYJ‐DZX‐02); Scientific Research Project of Guangzhou Education Bureau (Grant No. 202235395); Guangdong Basic and Applied Basic Research Foundation (Grant No. 2020A1515011048), and Basic Research Program of Guizhou Province: Guizhou Science and Technology Foundation (Grant No. ZK [2021] General 418).

## CONFLICT OF INTEREST

All authors claim that there are no conflicts of interest.

## INFORMED CONSENT

The patients gave their informed consents for this report.

## Supporting information


Appendix S1

## Data Availability

The data that support the findings of this study are available in the supplementary material of this article or available from the corresponding author upon reasonable request.
