# Supplementary material for: Variants in BRWD3 associated with X‐linked partial epilepsy without intellectual disability
Source: CNS Neurosci Ther. 2022 Dec 13;29(2):727–35. doi: 10.1111/cns.14057 (PMC9873514; doi:10.1111/cns.14057)
Supplement: Supplementary file 1 — Appendix S1 [file CNS-29-727-s001.docx]

**T**able S1. Summary of clinical features in the cases/families with *BRWD3* mutations

| **Mutations** | **Location** | **Gender** | **Phenotype** | **References** |
| --- | --- | --- | --- | --- |
| **Destructive mutations (21)** |  |  |  |  |
| **Nonsense mutations (9)** |  |  |  |  |
| c.451C>T; Gln151* | N-non | Male | MRX93 | (Tatton-Brown et al., 2017) |
| c.568C>T; Arg190* | WDrD1 | Male | MRX93 | (Tatton-Brown et al., 2017) |
| c.696T>A; Tyr232* | WDrD4 | Male | MRX93 | (Grozeva et al., 2015) |
| c.4487C>A; p. Ser1496* | C-non | Male | MRX93 | (Field et al., 2007) |
| c.2368C>T; p. Glu790* | B-non | Male | MRX93 | (Ostrowski et al., 2019) |
| c.3393C>G; p. Tyr1131* | B-non | Male | MRX93 | (Grotto et al., 2014) |
| c.3976C>T; p. Arg1326*# | BRD2 | Male | MRX93 | (Tatton-Brown et al., 2017) |
| c.3976C>T; p. Arg1326*# | BRD2 | Female | MRX93 | (Tatton-Brown et al., 2017) |
| c.4487C>A; p. Ser1496* | C-non | Male | MRX93 | (Ostrowski et al., 2019) |
| c.5080C>T; p. R1694* | C-non | Male | MRX93 | (Tenorio et al., 2019) |
| **Frame shift mutations (6)** |  |  |  |  |
| c.171delC; p. Phe57Leufs*27 | N-non | Male | MRX93 | (Ostrowski et al., 2019) |
| c.447_451del p. Arg150Ilefs*5 | N-non | Male | MRX93 | (Tatton-Brown et al., 2017) |
| c.946_947insA; p. Arg316Lysfs*21 | WDrD4 | Male | MRX93 | (Field et al., 2007) |
| c.1848delT; p. Ile617Tyrfs*14 | B-non | NA | MRX93 | (LaDuca et al., 2017) |
| c.2062_2064delinsCCAT; p. Met688Profs*2 | B-non | Male | MRX93 | (Tatton-Brown et al., 2017) |
| c.3791delG; p. Ser1264Ilefs*13 | B-non | Male | MRX93 | (Grozeva et al., 2015) |
| **Splice site mutations (3)** |  |  |  |  |
| c.1127+1G>A# | - | Male | MRX93 | (Tenorio et al., 2019) |
| c.1127+1G>A# | - | Female | MRX93 | (Ostrowski et al., 2019) |
| c.3325+1G>T | - | Male | MRX93 | (Field et al., 2007) |
| c.3602+1G>A | - | Male | MRX93 | (Ostrowski et al., 2019) |
| **Gross deletions (3)** |  |  |  |  |
| 80 kb | Exon 1-13 | Male | MRX93 | (Ostrowski et al., 2019) |
| 97 kb | Exon 8-41 | Male | MRX93 | (Grotto et al., 2014) |
| 74 kb (79915277-79989789) | Exon 11-41 | Male | MRX93 | (Ostrowski et al., 2019) |
| **Missense mutations (9)** |  |  |  |  |
| c.67G>C; p. Gly23Arg | N-non | Male | MRX93 | (Vissers et al., 2017) |
| c.256G>A; p. Glu86Lys | N-non | Male | MRX93 | (Gao et al., 2019) |
| c.836C>T; p. Thr279Ile+ | WDrD3 | Male | Seizure-controlled partial epilepsy without ID | Present study |
| c.836C>T; p. Thr279Ile+ | WDrD3 | Female | Two focal seizures triggered by fever | Present study |
| c.922C>G; p. Pro308Ala | WDrD4 | Male | Epilepsy (West syndrome) | (Hino-Fukuyo et al., 2015) |
| c.2824A>G; p. Met942Val | B-non | Male | Mild speech disorder | (Hildebrand et al., 2020) |
| c.3413G>T; p. Trp1138Leu# | B-non | Male | MRX93 | (Tenorio et al., 2019) |
| c.3413G>T; p. Trp1138Leu# | B-non | Female | MRX93 | (Tenorio et al., 2019) |
| c.4234A>C; p. Ile1412Leu+ | BRD2 | Male | Seizure-controlled partial epilepsy without ID, febrile seizure | Present study |
| c.4234A>C; p. Ile1412Leu+ | BRD2 | Male | Seizure-controlled partial epilepsy without ID, febrile seizure | Present study |
| c.4255T>G; p. Leu1419Val | C-non | Male | MRX93 | (Lee et al., 2014) |
| c.4786A>G; p. Lys1596Glu | C-non | Male | MRX93 | (Field et al., 2007) |
| Intronic mutations (2) |  |  |  |  |
| c.1877-5T>C | - | Male | Seizure-controlled epilepsy and ASD | (Long et al., 2019) |
| c.2475+6A>G | - | Male | Seizure-controlled partial epilepsy without ID | Present study |
| **Gross duplications (3)** |  |  |  |  |
| 690 kb (79368628-80059046) | Exon 5-41 | Female | Cognitive impairment | (Di Gregorio et al., 2017) |
| 177kb (79915477-80032646) | Exon 7-41 | Male | MRX93 and obesity | (Tassano et al., 2018) |
| 155kb (79975010-80130433) | Exon 18-41 | Male | Rolandic epilepsy | (Dimassi et al., 2014) |

Abbreviations: ASD, autism Spectrum Disorder BRD, Bromodomain; B-non, between WD and BRD; C-non, C-terminal nondomain; ID, intellectual disability; NA, not available; N-non, N-terminal nondomain; WDrD, WD40 repeat domain.

+ Recurrent mutations from present study. # Mutations from one family.

References

Di Gregorio, E., Riberi, E., Belligni, E. F., Biamino, E., Spielmann, M., Ala, U., . . . Ferrero, G. B. (2017). Copy number variants analysis in a cohort of isolated and syndromic developmental delay/intellectual disability reveals novel genomic disorders, position effects and candidate disease genes. *Clin Genet, 92*(4), 415-422. Retrieved from <https://www.ncbi.nlm.nih.gov/pubmed/28295210>. doi:10.1111/cge.13009

Dimassi, S., Labalme, A., Lesca, G., Rudolf, G., Bruneau, N., Hirsch, E., . . . Sanlaville, D. (2014). A subset of genomic alterations detected in rolandic epilepsies contains candidate or known epilepsy genes including GRIN2A and PRRT2. *Epilepsia, 55*(2), 370-378. Retrieved from <https://www.ncbi.nlm.nih.gov/pubmed/24372385>. doi:10.1111/epi.12502

Field, M., Tarpey, P. S., Smith, R., Edkins, S., O'Meara, S., Stevens, C., . . . Raymond, F. L. (2007). Mutations in the BRWD3 gene cause X-linked mental retardation associated with macrocephaly. *Am J Hum Genet, 81*(2), 367-374. Retrieved from <https://www.ncbi.nlm.nih.gov/pubmed/17668385>. doi:10.1086/520677

Gao, C., Wang, X., Mei, S., Li, D., Duan, J., Zhang, P., . . . Yang, X. A. (2019). Diagnostic Yields of Trio-WES Accompanied by CNVseq for Rare Neurodevelopmental Disorders. *Front Genet, 10*, 485. Retrieved from <https://www.ncbi.nlm.nih.gov/pubmed/31178897>. doi:10.3389/fgene.2019.00485

Grotto, S., Drouin-Garraud, V., Ounap, K., Puusepp-Benazzouz, H., Schuurs-Hoeijmakers, J., Le Meur, N., . . . Saugier-Veber, P. (2014). Clinical assessment of five patients with BRWD3 mutation at Xq21.1 gives further evidence for mild to moderate intellectual disability and macrocephaly. *Eur J Med Genet, 57*(5), 200-206. Retrieved from <https://www.ncbi.nlm.nih.gov/pubmed/24462886>. doi:10.1016/j.ejmg.2013.12.012

Grozeva, D., Carss, K., Spasic-Boskovic, O., Tejada, M. I., Gecz, J., Shaw, M., . . . Raymond, F. L. (2015). Targeted Next-Generation Sequencing Analysis of 1,000 Individuals with Intellectual Disability. *Hum Mutat, 36*(12), 1197-1204. Retrieved from <https://www.ncbi.nlm.nih.gov/pubmed/26350204>. doi:10.1002/humu.22901

Hildebrand, M. S., Jackson, V. E., Scerri, T. S., Van Reyk, O., Coleman, M., Braden, R. O., . . . Morgan, A. T. (2020). Severe childhood speech disorder: Gene discovery highlights transcriptional dysregulation. *Neurology, 94*(20), e2148-e2167. Retrieved from <https://www.ncbi.nlm.nih.gov/pubmed/32345733>. doi:10.1212/WNL.0000000000009441

Hino-Fukuyo, N., Kikuchi, A., Arai-Ichinoi, N., Niihori, T., Sato, R., Suzuki, T., . . . Kure, S. (2015). Genomic analysis identifies candidate pathogenic variants in 9 of 18 patients with unexplained West syndrome. *Hum Genet, 134*(6), 649-658. Retrieved from <https://www.ncbi.nlm.nih.gov/pubmed/25877686>. doi:10.1007/s00439-015-1553-6

LaDuca, H., Farwell, K. D., Vuong, H., Lu, H. M., Mu, W., Shahmirzadi, L., . . . Chao, E. C. (2017). Exome sequencing covers >98% of mutations identified on targeted next generation sequencing panels. *PLoS One, 12*(2), e0170843. Retrieved from <https://www.ncbi.nlm.nih.gov/pubmed/28152038>. doi:10.1371/journal.pone.0170843

Lee, H., Deignan, J. L., Dorrani, N., Strom, S. P., Kantarci, S., Quintero-Rivera, F., . . . Nelson, S. F. (2014). Clinical exome sequencing for genetic identification of rare Mendelian disorders. *JAMA, 312*(18), 1880-1887. Retrieved from <https://www.ncbi.nlm.nih.gov/pubmed/25326637>. doi:10.1001/jama.2014.14604

Long, S., Zhou, H., Li, S., Wang, T., Ma, Y., Li, C., . . . Wang, Y. (2019). The Clinical and Genetic Features of Co-occurring Epilepsy and Autism Spectrum Disorder in Chinese Children. *Front Neurol, 10*, 505. Retrieved from <https://www.ncbi.nlm.nih.gov/pubmed/31139143>. doi:10.3389/fneur.2019.00505

Ostrowski, P. J., Zachariou, A., Loveday, C., Baralle, D., Blair, E., Douzgou, S., . . . Tatton-Brown, K. (2019). Null variants and deletions in BRWD3 cause an X-linked syndrome of mild-moderate intellectual disability, macrocephaly, and obesity: A series of 17 patients. *Am J Med Genet C Semin Med Genet, 181*(4), 638-643. Retrieved from <https://www.ncbi.nlm.nih.gov/pubmed/31714006>. doi:10.1002/ajmg.c.31750

Tassano, E., Uccella, S., Giacomini, T., Striano, P., Severino, M., Porta, S., . . . Ronchetto, P. (2018). Intragenic Microdeletion of ULK4 and Partial Microduplication of BRWD3 in Siblings with Neuropsychiatric Features and Obesity. *Cytogenet Genome Res*. Retrieved from <https://www.ncbi.nlm.nih.gov/pubmed/30086552>. doi:10.1159/000491871

Tatton-Brown, K., Loveday, C., Yost, S., Clarke, M., Ramsay, E., Zachariou, A., . . . Rahman, N. (2017). Mutations in Epigenetic Regulation Genes Are a Major Cause of Overgrowth with Intellectual Disability. *Am J Hum Genet, 100*(5), 725-736. Retrieved from <https://www.ncbi.nlm.nih.gov/pubmed/28475857>. doi:10.1016/j.ajhg.2017.03.010

Tenorio, J., Alarcon, P., Arias, P., Ramos, F. J., Campistol, J., Climent, S., . . . Lapunzina, P. (2019). MRX93 syndrome (BRWD3 gene): five new patients with novel mutations. *Clin Genet, 95*(6), 726-731. Retrieved from <https://www.ncbi.nlm.nih.gov/pubmed/30628072>. doi:10.1111/cge.13504

Vissers, L., van Nimwegen, K. J. M., Schieving, J. H., Kamsteeg, E. J., Kleefstra, T., Yntema, H. G., . . . Willemsen, M. (2017). A clinical utility study of exome sequencing versus conventional genetic testing in pediatric neurology. *Genet Med, 19*(9), 1055-1063. Retrieved from <https://www.ncbi.nlm.nih.gov/pubmed/28333917>. doi:10.1038/gim.2017.1
